# Supplementary material for: Discovery of a small molecule that selectively destabilizes Cryptochrome 1 and enhances life span in p53 knockout mice
Source: Nat Commun. 2022 Nov 8;13:6742. doi: 10.1038/s41467-022-34582-1 (PMC9643396; doi:10.1038/s41467-022-34582-1)
Supplement: Supplementary file 1 — Supplementary Information [file 41467_2022_34582_MOESM1_ESM.pdf]

Supplementary Information

Discovery of a small molecule that selectively destabilizes Cryptochrome 1 and enhances life span in p53 knockout mice

Seref Gul[1#], Yasemin Kubra Akyel[2э], Zeynep Melis Gul[3], Safak Isin[3], Onur Ozcan[3], Tuba Korkmaz[4], Saba Selvi[4], Ibrahim Danis[5,6], Ozgecan Savlug Ipek[7,8], Fatih Aygenli[4], Ali Cihan Taskin[9], Büşra Aytül Akarlar[3], Nurhan Ozlu[3], Nuri Ozturk[4], Narin Ozturk[2], Durişehvar Özer Ünal[5,6], Mustafa Guzel[7,10], Metin Turkay[11], Alper Okyar[2], Ibrahim Halil Kavakli[1,3*]

*Corresponding author: e-mail address: hkavakli@ku.edu.tr

Supplementary Data:

1. Supplementary (Fig. 1-4).
2. Supplemental Tables (Table S1).

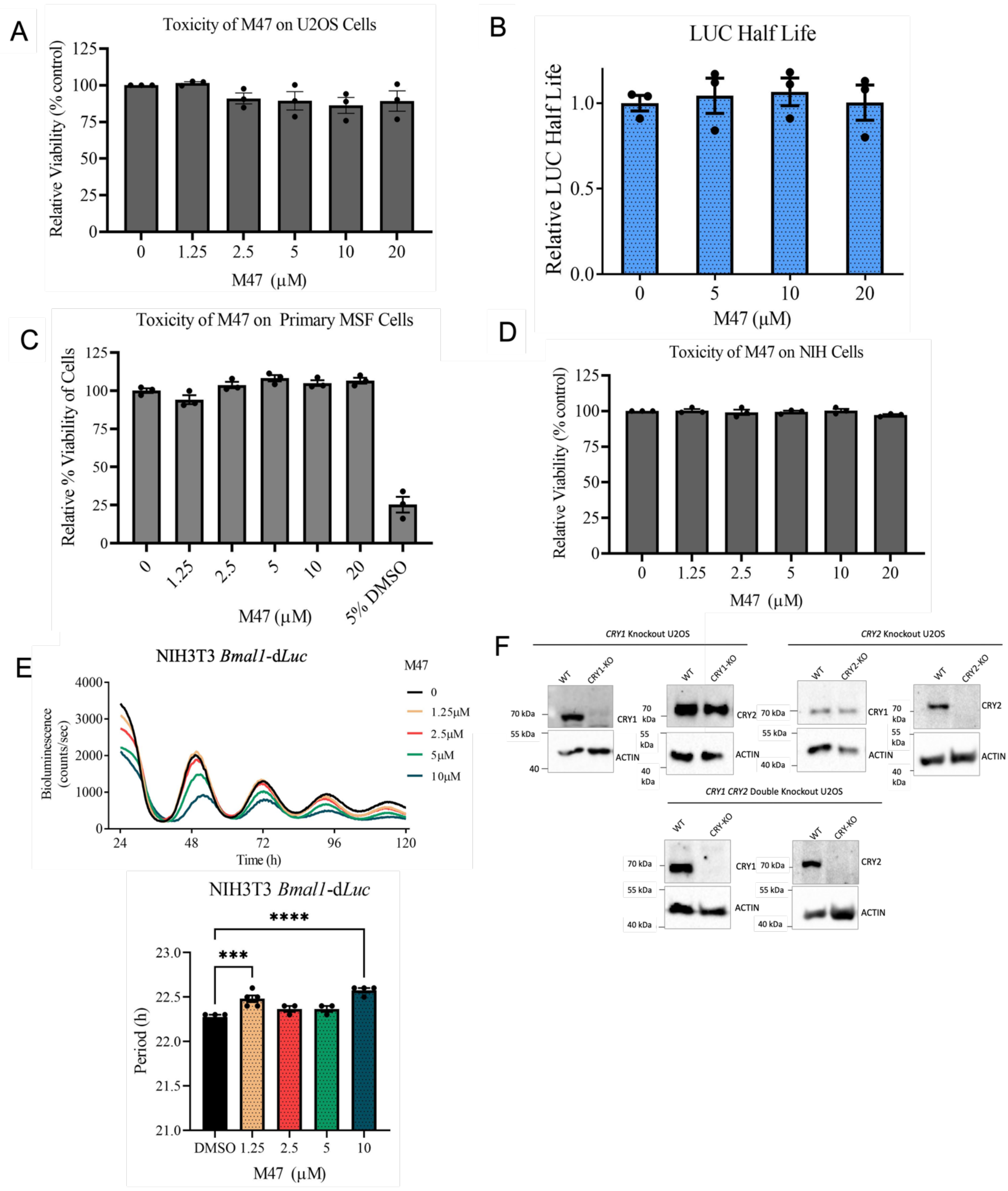

Fig. S1. Dose dependent effect of M47 cell viability, LUC degradation and NIH3T3 cells, CRISPR/Cas9 CRY1 deletion confirmation. (**A**) Cytotoxicity of M47 doses on U2OS cells were tested by the MTT assay (Data represent the mean ± SEM, n=3 with triplicates). (**B**) M47 did not interfere with the degradation rate of LUC itself. LUC degradation assay was mainly performed as explained in Fig. S3A, however, instead of 40ng *Cry-Luc*, 5ng d*Luc* plasmid was transfected due to its high level of expression and luminescence (Data represent the mean ± SEM, n=3 with triplicates with one-way ANOVA). (**C**) Cytotoxicity of M47 doses on primary mouse fibroblast (MSF) cells were tested by the MTT assay (Data represent

the mean ± SEM, n=3 with triplicates). (**D**) Cytotoxicity of M47 doses on NIH 3T3 cells (Data represent the mean ± SEM, n=3 with triplicates). (**E**) Dose dependent effect of M47 was tested on the NIH3T3 cells stably expressing *Bmal1-dLuc* **p=0.0036, ***p=0.0001 (n=4± SEM with one-way ANOVA with Dunnet's multiple comparison test). (**F**) Confirmation of CRY knockouts in the U2OS cell line. Immunoblot of CRY1 and CRY2 proteins in the CRY2 knockout U2OS clone indicated as CRY2 knockout U2OS. Immunoblot of CRY1 and CRY2 in the CRY1&CRY2 double knockout U2OS clones indicated as CRY1CRY2 double knockout U2OS. Confirmation of CRY1 knockout in the U2OS cell line. Immunoblot of CRY1 and CRY2 proteins in the U2OS clone was used to confirm that specific knockout of CRY1 was successful (n=3). Actin was blotted as the loading control. Numbers on the left of each panel indicate the positions of the corresponding molecular size markers in kDa. WT: Wildtype; KO: Knockout.

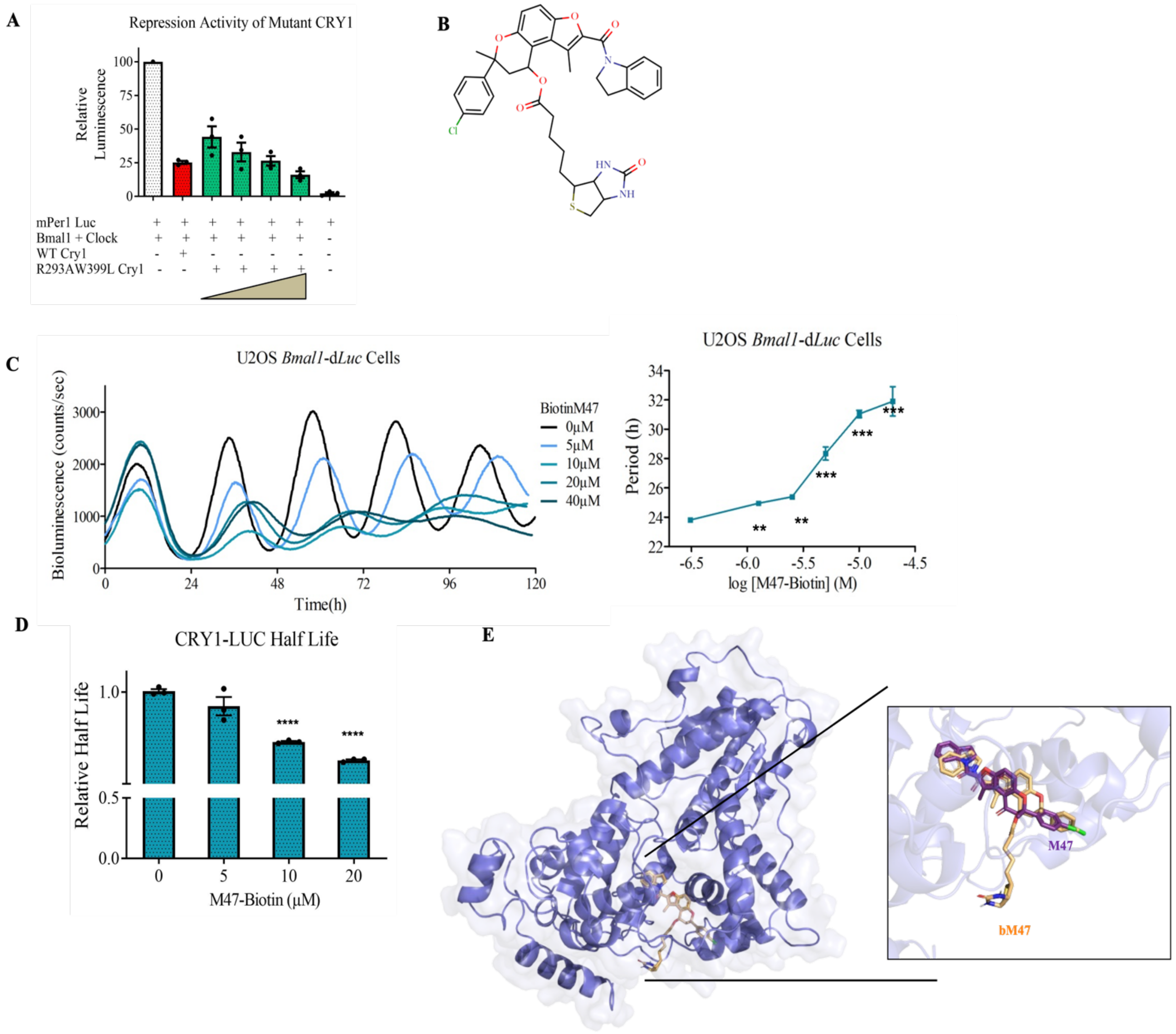

Fig. S2. Activity of mutant CRY and effect of biotin on the activity of M47. (**A**) Mutant R293AW399L CRY1 was active and could repress the BMAL1/CLOCK mediated transcription. Wildtype CRY1 was used as the positive control. Data were normalized according to transcription activity of BMAL1/CLOCK without ectopic expression of CRY1 (Data represent the mean ± SEM, n=3 with triplicates). (**B**) Chemical structure of bM47 used for pull-down assays. (**C**) Biotin attached to M47 did not change the effect of M47 on the circadian rhythm. Luminescence data is a representative of two independent (n=2) experiments with duplicates. Period data is reported as the mean ± SEM, n=2 with duplicates ***p=0.0001, **p=0.0078versus DMSO control by one way ANOVA with Dunnet's multiple comparison test. (**D**) bM47 enhanced the degradation rate of CRY1-LUC. Data represent the mean ±SEM, n=3 with triplicates ****p < 0.0001 versus DMSO control by one way ANOVA with Dunnet's multiple comparison test. (**E**) Binding modes of bM47 and M47 on primary packet of CRY1. Yellow color is bM47 while purple color is M47.

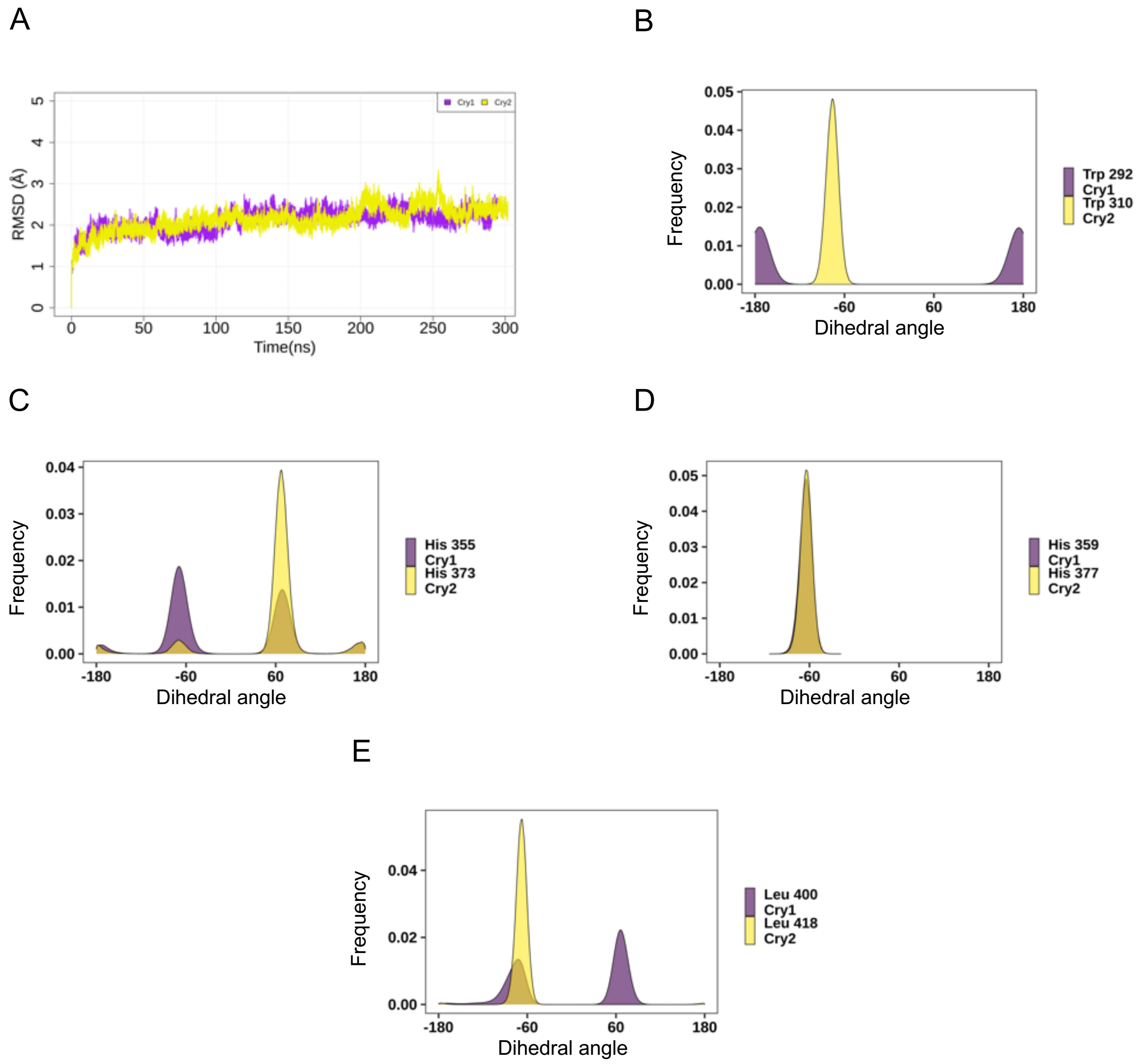

Fig. S3. Molecular dynamic simulation of Cryptochromes. (A) RMSD values of CRY1 and CRY2 PHR domain. (B-E) χ_1(Chi-1) dihedral angle distributions of primary pocket residues in Cry1 and Cry2 throughout MD simulations.

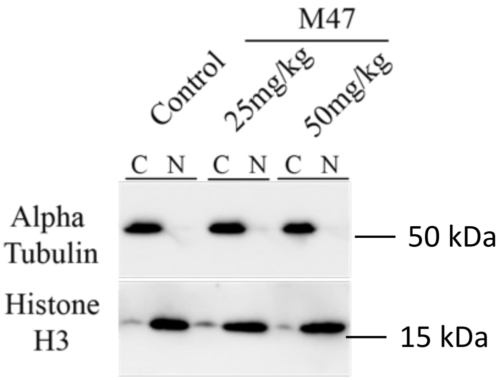

Fig. S4. Subcellular fractionation control of mice liver. Liver samples were fractionated as cytosolic (C) and nuclear (N) parts. Alpha tubulin and Histone H3 were probed as the cytosolic and nuclear protein markers, respectively (n=3).

Table S1. Sequence of primers for Cry-Luc cloning, site-directed mutagenesis and RT-qPCR.

Luc-pcDNA-NotI-F	GCACAGTGGCGGCCGCTCATGGAAGACGCCAAAAAC
Luc-pcDNA-XhoI-R	TCTAGACTCGAGCACGGCGATCTTTCC
mCRY1-pcDNA-EcoRV-F	TTCTGCAGATATCCAATGGGGGTGAACGCCGTG
mCRY1-pcDNA-NotI-R	AGACTCGAGCGGCCGCCAGTTACTGCTCTGCCGCTG
mCRY2-pcDNA-EcoRV-F	TTCTGCAGATATCCAATGGCGGCGGCTGCTGTG
mCRY2-pcDNA-NotI-R	GAGCGGCCGCCACTGTGCGGAGTCCTTGCTTGCTGG
mCRY1_W399L_F	GCTGGAAGTTGGATGTTGCTGTCCTGCAGTTCC
mCRY1_W399L_R	CGACCTTCAACCTACAACGACAGGACGTCAAGG
mCRY1_R293A_F	CTTTATGGGCAACTCCTGTGGGCTGAATTTTTTATACAGCAG
mCRY1_R293A_R	CTGCTGTATAAAAAATTCAGCCCACAGGAGTTGCCCATAAAG
RT_*hBMAL1*_F	GCCCATTGAACATCACGAGTAC
RT_*hBMAL1*_R	CCTGAGCCTGGCCTGATAGTAG
RT_*hCRY1*_F	ACAGGTGGCGATTTTTGCTTC
RT_*hCRY1*_R	TCCAAAGGGCTCAGAATCATACT
RT_*hCRY2*_F	CTACCGGGGACTCTGTCTACT
RT_*hCRY2*_R	ACTGGGTAGTGGTCTTGGGC
RT_*hDBP*_F	GAGGAACTTAAGCCCCAGCC
RT_*hDBP*_R	CTCGTTGTTCTTGTACCGCC
RT_*hPER2*_F	GCGTGTTCCACAGTTTCACC
RT_*hPER2*_R	GGCTTTTCCGGACACTGACA
RT_*hGAPDH*_F	TGCACCACCAACTGCTTAGC
RT_*hGAPDH*_R	ACAGTCTTCTGGGTGGCAGTG
RT_*mGapdh*_F	AACTTTGGCATTGTGGAAGG
RT_*mGapdh*_R	ACACATTGGGGGTAGGAACA
RT_*mPer2*_F	GAGCGCCACCAAGTGACGG
RT_*mPer2*_R	GGTGGGACTTGGGGAGAAGT
RT_*p53*_F	
RT_*p53*_R	
