# Supplementary material for: Discovery of a small molecule that selectively destabilizes Cryptochrome 1 and enhances life span in p53 knockout mice
Source: Nat Commun. 2022 Nov 8;13:6742. doi: 10.1038/s41467-022-34582-1 (PMC9643396; doi:10.1038/s41467-022-34582-1)
Supplement: Supplementary file 3 — Reporting Summary [file 41467_2022_34582_MOESM3_ESM.pdf]

nature portfolio

Corresponding author(s): Ibrahim Halil Kavakli

Last updated by author(s): 30/09/22

Reporting Summary

Nature Portfolio wishes to improve the reproducibility of the work that we publish. This form provides structure for consistency and transparency in reporting. For further information on Nature Portfolio policies, see our Editorial Policies and the Editorial Policy Checklist.

Statistics

For all statistical analyses, confirm that the following items are present in the figure legend, table legend, main text, or Methods section.

n/a	Confirmed	
[ ]	[x]	The exact sample size (n) for each experimental group/condition, given as a discrete number and unit of measurement
[ ]	[x]	A statement on whether measurements were taken from distinct samples or whether the same sample was measured repeatedly
[ ]	[x]	The statistical test(s) used AND whether they are one- or two-sided<br>*Only common tests should be described solely by name; describe more complex techniques in the Methods section.*
[x]	[ ]	A description of all covariates tested
[ ]	[x]	A description of any assumptions or corrections, such as tests of normality and adjustment for multiple comparisons
[ ]	[x]	A full description of the statistical parameters including central tendency (e.g. means) or other basic estimates (e.g. regression coefficient) AND variation (e.g. standard deviation) or associated estimates of uncertainty (e.g. confidence intervals)
[ ]	[x]	For null hypothesis testing, the test statistic (e.g. F, t, r) with confidence intervals, effect sizes, degrees of freedom and P value noted<br>*Give P values as exact values whenever suitable.*
[x]	[ ]	For Bayesian analysis, information on the choice of priors and Markov chain Monte Carlo settings
[x]	[ ]	For hierarchical and complex designs, identification of the appropriate level for tests and full reporting of outcomes
[x]	[ ]	Estimates of effect sizes (e.g. Cohen's d, Pearson's r), indicating how they were calculated

Our web collection on statistics for biologists contains articles on many of the points above.

Software and code

Policy information about availability of computer code

Data collection	LumiCycle software, VMD 1.9.1, NAMD 2.13, Autodock vina v.1.2, Autodock4.2, Autodock Tools4, Open Babel 2.4.1, Thermo Proteome Discoverer versions 1.4 and 2.3, Synergy H1 (Biotek), ChemiDoc XRS+ system (Bio-Rad), RaptorX webserver, CHARMM-PARAM22,force field, BioRad ChemiDoc Touch
Data analysis	LumiCycle Analysis v3.002, Biorad image Lab 6.1, Biorad CFX manger 3.1, VMD 1.9.1, NAMD 2.13, Autodock vina v.1.2, Autodock4.2, Autodock Tools4, PymoL 2.5.0 (open source), Thermo Proteome Discoverer versions 1.4 and 2.3, R-Program 6.3, RaptorX webserver, UCSF Chimera v.1.14, GraphPad Prism v.5.0 ,GraphPad Prism v.8.0

For manuscripts utilizing custom algorithms or software that are central to the research but not yet described in published literature, software must be made available to editors and reviewers. We strongly encourage code deposition in a community repository (e.g. GitHub). See the Nature Portfolio guidelines for submitting code & software for further information.

Data

Policy information about availability of data

All manuscripts must include a data availability statement. This statement should provide the following information, where applicable:

- Accession codes, unique identifiers, or web links for publicly available datasets
- A description of any restrictions on data availability
- For clinical datasets or third party data, please ensure that the statement adheres to our policy

All data needed to evaluate the conclusions in the paper are present in the paper and/or the Supplementary Materials. Additional data related to this paper may be

requested from the authors. Mouse CRY1 (PDB ID: 4K0R), mouse CRY2 (PDB ID: 4I6G),

Human research participants

Policy information about studies involving human research participants and Sex and Gender in Research.

Reporting on sex and gender	N/A
Population characteristics	N/A
Recruitment	N/A
Ethics oversight	N/A

Note that full information on the approval of the study protocol must also be provided in the manuscript.

Field-specific reporting

Please select the one below that is the best fit for your research. If you are not sure, read the appropriate sections before making your selection.

☒ Life sciences ☐ Behavioural & social sciences ☐ Ecological, evolutionary & environmental sciences

For a reference copy of the document with all sections, see nature.com/documents/nr-reporting-summary-flat.pdf

Life sciences study design

All studies must disclose on these points even when the disclosure is negative.

Sample size	We did not carry out predetermination of sample size. Where required for statistical analyses, sample size included at least three independent biological replicates. In some cases, the number of n is more than 3 dependent on available material and number of samples can be processed reliably in parallel. We concluded that the sample size is enough to cover the biological variance when the data was highly reproducible.
Data exclusions	No data were excluded
Replication	For each experiments at least three independent and successful replicates were provided.
Randomization	Allocation of animals were random . For in vitro studies, conditions were randomized into control and experimental conditions as described in each assay.
Blinding	Investigators were not blinded during experiments other than LC-MS, because no subjective process is included in all the analyses of the experimental data in this study. LS-MS experiments were performed in facility where samples were just numbered as 1, 2, 3, and 4. They run and analyzed blindly.

Reporting for specific materials, systems and methods

We require information from authors about some types of materials, experimental systems and methods used in many studies. Here, indicate whether each material, system or method listed is relevant to your study. If you are not sure if a list item applies to your research, read the appropriate section before selecting a response.

Materials & experimental systems

n/a	Involved in the study	
☐	☒	Antibodies
☐	☒	Eukaryotic cell lines
☒	☐	Palaeontology and archaeology
☐	☒	Animals and other organisms
☒	☐	Clinical data
☒	☐	Dual use research of concern

Methods

n/a	Involved in the study	
☒	☐	ChIP-seq
☒	☐	Flow cytometry
☒	☐	MRI-based neuroimaging

Antibodies

Antibodies used	Cry1 Antibody: Bethyl (A302-614A);Cry2 Antibody: Bethyl (A302-615A);GAL4 (DBD) antibody: Santa Cruz (SC-577); Histone H3 antibody: abcam (ab1791); Alpha Actin antibody : Sigma (Sigma T9026); His Antibody: Santa Cruz (sc-8036); Beta-Actin antibody

	mouse: Cell Signaling (8H10D10); m-IgGκ BP-HRP: Santa Cruz (sc-516102), anti-rabbit IgG-HRP: Santa Cruz (sc-2357); CLOCK antibody:Bethyl (A302-618A); MYC antibody:abcam (ab18185); BMAL1 antibody: Santa Cruz (sc365645)
Validation	anti-Cry2, https://www.bethyl.com/product/A302-615A?referrer=search; Anti-GAL4, http://datasheets.scbt.com/sc-577.pdf; Anti Histone H3, https://www.abcam.com/histone-h3-antibody-nuclear-loading-control-and-chip-grade-ab1791.html; Anti-Alpha Actin, https://www.sigmaaldrich.com/catalog/product/sigma/t9026?lang=en&region=TR; Anti-Actin, https://www.cellsignal.com/products/primary-antibodies/b-actin-8h10d10-mouse-mab/3700; Anti His, (http://datasheets.scbt.com/sc-8036.pdf); Anti mouse IgG-HRP, https://www.scbt.com/scbt/product/m-igg-kappa-bp-hrp; Anti rabbit-HRP, https://www.scbt.com/scbt/product/mouse-anti-rabbit-igg-hrp; Clock antibody (https://www.biomol.com/products/antibodies/primary-antibodies/general/anti-clock-a302-618a-t); BMAL1 antbody (https://www.scbt.com/p/bmal1-antibody-b-1), Anti-PARP (https://www.cellsignal.com/products/primary-antibodies/parp-antibody/9542); Anti-Myc (https://www.abcam.com/myc-tag-antibody-myca7-ab18185.html)

Eukaryotic cell lines

Policy information about cell lines and Sex and Gender in Research

Cell line source(s)	HEK 293T (ATTC CRL-3216): U2OSBmal1-dLuc and NIH3T3 Per1-dLuc from Prof John Hogenecsh (Cincinnati Children Hospital, USA), Mouse Skin Fibroblast (MSF) cell Line from Aziz Sancar Lab
Authentication	We didn't make addition effort for cell line authentication, since they were obtained either from ATCC or from Dr. John Hogenesch (Cincinnati Children Hospital, USA) who generated the cell lines and from Aziz Sancar Lab (University of North Caroline-Chapel Hill.
Mycoplasma contamination	The cell lines were tested for mycoplasma contamination using Mycoalert mycoplasma detection Kit from Lonza (LT07-318). The results were negative.
Commonly misidentified lines (See ICLAC register)	Commonly misidentified cell lines were not used

Animals and other research organisms

Policy information about studies involving animals; ARRIVE guidelines recommended for reporting animal research, and Sex and Gender in Research

Laboratory animals	p53+/- transgenic mice were purchased form Jackson Laboratory (Stock number:002101) and breded in house to obtain p53-/-. Then mutant animals were used in the survival experiments in the age of 4-6 weeks. C57BL/6J mice were purchased form Jackson Laboratory (Stock number:000664).
Wild animals	The study did not involve wild animals.
Reporting on sex	PK experiments were done on females. Survival rate were calculated from male p53 mutant animals.
Field-collected samples	No field collected samples were used in the study.
Ethics oversight	experiments were conducted in accordance with the guidelines approved for animal experimental procedures by the Koc University Animal Research Local Ethics Committee (No: 2015/13)

Note that full information on the approval of the study protocol must also be provided in the manuscript.
